# Study on Adult Chinchilla (*Chinchilla lanigera*) Preferences for Cages of Different Sizes

**DOI:** 10.3390/ani14233368

**Published:** 2024-11-22

**Authors:** Zsolt Szendrő, Stanisław Łapiński, Zsolt Matics, Zsolt Gerencsér

**Affiliations:** 1 Institute of Physiology and Nutrition, Kaposvár Campus, Hungarian University of Agriculture and Life Sciences, 7400 Kaposvár, Hungary; 2 Department of Zoology and Animal Welfare, University of Agriculture in Krakow, 31120 Kraków, Poland; stanislaw.lapinski@urk.edu.pl; 3 Department of Animal Sciences, Széchenyi István University, 9200 Mosonmagyaróvár, Hungary; matics.zsolt@ga.sze.hu; 4 Institute of Animal Sciences, Kaposvár Campus, Hungarian University of Agriculture and Life Sciences, 7400 Kaposvár, Hungary; gerencser.zsolt@uni-mate.hu

**Keywords:** *Chinchilla lanigera*, housing, preference test, welfare

## Abstract

The study aimed to investigate adult chinchillas’ preferences when choosing between cages with different floor areas, heights, or both. The size of the cages with a larger floor area was double that of the standard-sized cages in the farms (0.25 and 0.50 m^2^), while their height was 2.4 times that of the standard-sized cages (0.4 and 1 m). The chinchillas (10–14 in each cohort) were housed in blocks of cages where they could move freely between the smaller and larger cages. The chinchillas’ location preferences were monitored continuously over five days using infrared video recording. The chinchillas were observed in the cages with smaller floor areas 1.9 and 2.7 times more frequently than in the larger ones (*p* < 0.001) during the dark (active) and light (resting) periods of the day, respectively. When the cage height was 0.4 m, they chose it 3.4 and 6.7 times more frequently, respectively, than the cage that was 1 m high (*p* < 0.001). When both the floor size and the height were increased, the chinchillas still favored the smaller and lower cages (*p* < 0.001). The study findings suggest that adult chinchillas show a preference for smaller and shorter cages when the small and large cages are not environment enrichment barren, especially during the rest period of the day, even when the floor area, height, or both are increased. In the future, it would be important to investigate different forms of cage enrichment.

## Simple Summary

The authors investigated what size cage adult chinchillas preferred when given a free choice. Compared to the cages traditionally used in chinchilla farms, the tested cages had a larger floor area, higher height, or both. It was found that the chinchillas chose cages with smaller floor areas and lower heights more often. Especially during the resting period, there was a significant difference between the choice of cages. The explanation for the results is that the wild chinchilla hides from predators in a narrow place, usually seeking shelter between rocks. Chinchillas in farms have retained this behavior and prefer smaller, more protected cages.

## 1. Introduction

**Figure 1:**
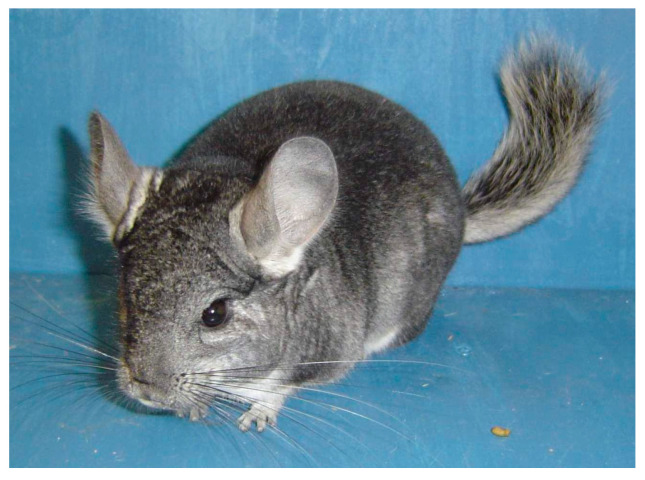
Adult chinchilla (*Chinchilla lanigera*) (Photo: Stanisław Łapiński).

Wild *Chinchilla lanigera* (long-tailed chinchilla (Figure 1), populations used to inhabit the Andes mountainous regions of Bolivia, Peru, and Argentina. However, the remaining colonies now reside in north-central Chile, specifically at elevations between 400 and 1650 m above sea level [1,2,3]. Their typical habitat is rocky or sandy terrain with sparse vegetation, which includes thorny shrubs, cacti, and bromeliads. Chinchillas are nocturnal and rarely leave their dens before sunset [4]. They typically seek shelter in rocky crevices, burrow beneath rocks, or shelter among large bromeliads to protect themselves from predators such as hawks, owls, wild cats, and wild dogs.

Chinchillas are primarily crepuscular and nocturnal. They sleep in shaded or concealed areas during the day and are active at dusk and at night, when the temperature is cool. Their dense fur provides insulation against the harsh climate of their high-altitude habitats. Wild chinchilla populations were commercially hunted for their valuable pelts, leading to their near extinction by the end of the 19th century [5]. Modern captive chinchillas are descended from 12 wild Chilean chinchillas captured in 1923 by Matthew Chapman [6]. In addition to fur production, chinchillas are used as animal models in medical research and as pets [7,8].

Farmed chinchillas are typically housed in polygamous cage systems with 4–6 females per male. Each female is housed in an individual cage, while the males have access to a corridor connected to the females’ cages. The females wear neck collars to prevent them from leaving their cages. Chinchillas are fed on commercial complete pelleted feed, and hay and dust baths are provided for cleaning their fur [9]. In most European chinchilla farms, the cage dimensions are approximately 0.4–0.5 m in depth, 0.5 m in width, and 0.34–0.4 m in height, while according to EU recommendations, cages should be 0.5 m in depth, 1.0 m in width, and 1.0 m in height [10,11].

Research has focused on developing welfare protocols for various farmed animals, but limited research has been conducted on chinchilla housing and behavior. Fur-chewing, a common issue in chinchilla farming, is associated with stress, which is often exacerbated by unsuitable housing conditions, and can lead to heat loss, affecting feed and water intake [12,13]. Studies have suggested that larger cages with environmental enrichment may reduce fearfulness and improve chinchilla welfare, highlighting the impact of cage complexity on behavior [10]. While genetic predispositions may also play a role in chinchilla behavior and welfare, current research focuses on the influence of cage size and enrichment [10,12].

This study aimed to investigate adult chinchillas’ preferences between different-sized cages (floor area, height, or both).

## 2. Materials and Methods

The experiment was conducted at Wanger Ltd.’s chinchilla farm in Komárom, Hungary. The experimental room was equipped with wire-mesh cages of different sizes, the floor was also wire mesh. Microclimate conditions were similar to those where the animals stayed on the farm before the test (average temperature: 19 °C and humidity: 65%). Lighting was provided for nine hours daily (7:00–16:00).

The chinchillas received commercial chinchilla complete pelleted feed produced by Wanger Ltd. (19% crude protein, 12% crude fiber, and 11.3 DE MJ/kg feed). Feed and water were provided ad libitum.

For each trial, 10–14 standard adult female chinchillas were randomly selected from the herd. Chinchillas could move freely between cages through a small hole in the walls. The preference tests were carried out in barren cages, without environment enrichment (e.g., no box, self, gnawing material, etc.). All cages were equipped with feeders and drinkers, and the only variable was the size of the cages (floor area, height, or both).

### 2.1. Cage Blocks with Different Floor Areas

Chinchillas could choose between two small cages and one larger cage, all with the same total floor area. Each small cage had a floor area of 0.25 m^2^, and the larger cage had a floor area of 0.5 m^2^. The height of the cages was the same within each group.

#### 2.1.1. Height of the Cage Block: 0.4 m

**Figure 2:**
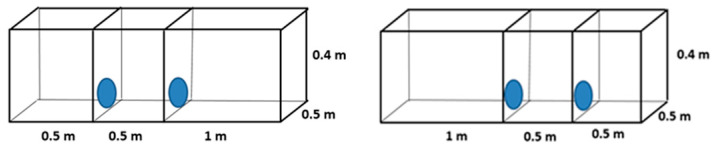
Design of cage blocks for preference testing of adult chinchillas, comparing 0.4 m high cages with different floor areas (0.25 and 0.5 m^2^).

The floor area of the large cage was 0.5 m^2^, while that of each small cage was 0.25 m^2^. The height of the cages was 0.4 m (Figure 2). The number of chinchillas was 13, evaluated over 65 (13 × 5) days.

#### 2.1.2. Height of the Cage Block: 1.0 m

**Figure 3:**
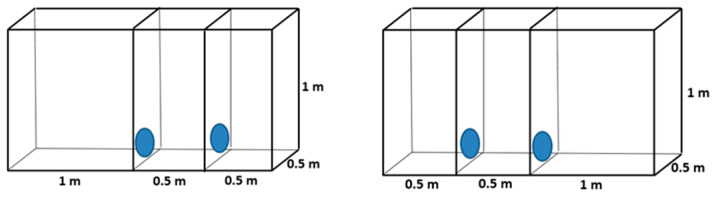
Design of cage blocks for preference testing of adult chinchillas, comparing 1 m high cages with different floor areas (0.25 and 0.5 m^2^).

The floor area of the large cage was 0.5 m^2^, and that of each small cage was 0.25 m^2^. The height of the cages was 1 m (Figure 3). The number of chinchillas was 13, evaluated over 65 (13 × 5) days.

### 2.2. Preference for Cages with Different Heights

Chinchillas were given a choice between two cages with different heights (0.4 m vs. 1 m) but with the same floor area.

#### 2.2.1. Floor Area: 0.25 m^2^

**Figure 4:**
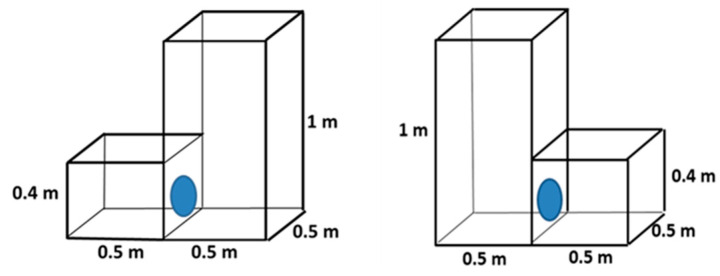
Design of cage blocks for preference testing of adult chinchillas, comparing cages with different heights and 0.25 m^2^ floor area.

Both cages had a floor area of 0.25 m^2^. One cage was 0.4 m high, and the other was 1 m high (Figure 4). The number of chinchillas was 14, evaluated over 70 (14 × 5) days.

#### 2.2.2. Floor Area: 0.5 m^2^

**Figure 5:**
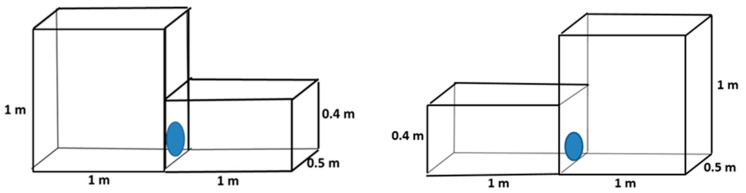
Design of cage blocks for preference testing of adult chinchillas, comparing cages with different heights and 0.5 m^2^ floor size.

Both cages had a floor area of 0.5 m^2^. One cage was 0.4 m high, and the other was 1 m high (Figure 5). The number of chinchillas was 14, evaluated over 70 (14 × 5) days.

### 2.3. Cages with Different Floor Areas and Heights

**Figure 6:**
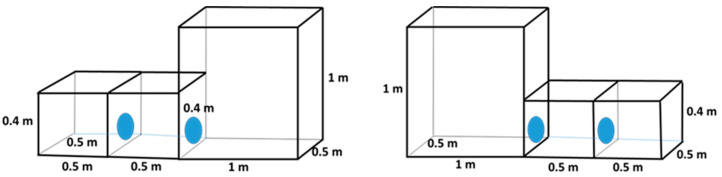
Design of cage blocks for preference testing of an adult chinchilla, comparing small and low, and large and high cages.

In this trial, a standard cage (0.25 m^2^ and 0.4 m high) and a larger cage (0.5 m^2^ and 1 m high) were compared (Figure 6). There were 10 chinchillas in each cage, and we evaluated them over a period of 50 (10 × 5) days.

One day before the preference test, a chinchilla was placed in a cage block (24 h acclimatization period), and we observed it for 5 days using continuous 24 h video recording. The infrared camera was inserted in front of the cage block. During recording, every 30 min (48 times a day) we recorded which cage the chinchilla was in (scan sampling).

The two scorers were not blind to treatment. The evaluation of the videos was straightforward (which cage the chinchilla was in), therefore, the results are reliable.

### 2.4. Statistical Analysis

The data were analyzed using the Chi-squared test in SPSS 10.0.

## 3. Results

The experiment involved varying the size of the cages in three different ways: by increasing the floor area, by increasing the height, or by altering both the floor area and height. The chinchillas were tested in each scenario. First, their preferences were assessed when the floor area was doubled. Next, their locations were examined when the floor area remained constant, but the height was increased. Lastly, their choices were observed when both the floor area and height were increased.

### 3.1. Preference for Cages with Different Floor Areas

The cage blocks were divided into two parts: one large cage, which was 0.5 m^2^ and made up 50% of the total floor area, and two small cages, each 0.25 m^2^, which together comprised the remaining 50%. During each observation, we recorded whether the chinchillas were staying in the large or small cages.

#### 3.1.1. Height of the Cage Block: 0.4 m

There were significant differences in the chinchillas’ cage preferences. On average, they spent 32% of their time in the large cage and 68% in the small cages (*p* < 0.001) over the five-day monitoring period. There were no significant differences in cage preference between observation days, with the small cages being chosen 65–71% of the time and the large cages 29–35% of the time.

**Figure 7:**
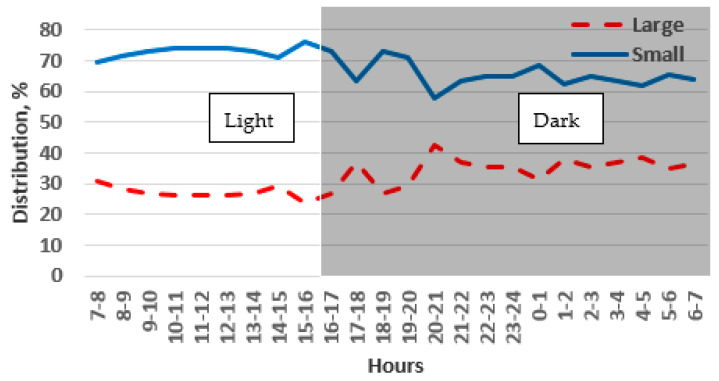
Preference of adult chinchillas for cages with a large floor area (0.5 m^2^) versus a small floor area (0.25 m^2^), both 0.4 m high, at different times of the day.

Throughout the monitoring period, there was a noticeable difference in the preference of the chinchillas between the light and dark phases of the day. During the light phase, the chinchillas chose the large cage 27% of the time, and this preference increased slightly to 35% during the dark phase. However, the chinchillas stayed most frequently in the small cages, with a preference of 73% during the dark phase and 65% during the light phase. These differences were statistically significant for each phase (*p* < 0.001). During the light phase, the chinchillas showed a preference for small cages ranging between 70% and 75% (Figure 7). However, during the dark phase, when the chinchillas were more active, the preference for small cages decreased slightly to a range of 60% to 65%.

#### 3.1.2. Height of the Cage Block: 1.0 m

**Figure 8:**
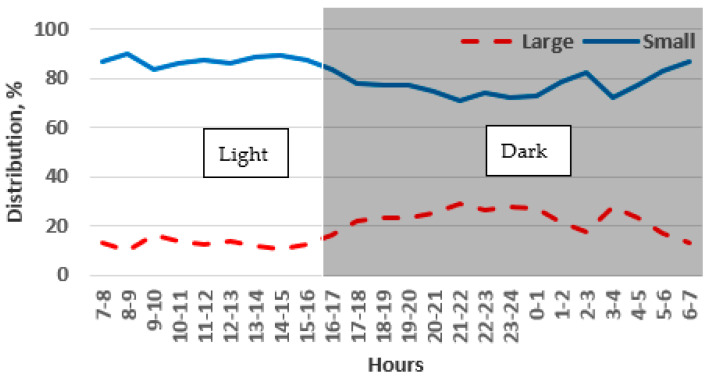
Preference of adult chinchillas for cages with a large floor area (0.5 m^2^) versus a small floor area (0.25 m^2^), both 1 m high, based on time of day.

In the 1.0 m high cage blocks, the chinchillas showed a preference for small cages 81% of the time and large cages 19% of the time (*p* < 0.001). The preference for a small cage was higher compared to the results for the 0.4 m high cage block (Figure 7 and Figure 8). Over the monitoring period, there was a slight decrease in the preference for small cages from 87% to 73% from day 1 to day 5, while the preference for large cages increased from 17% to 27%.

**Figure 9:**
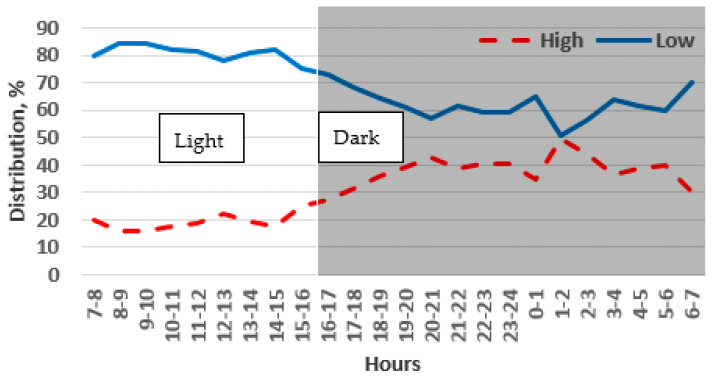
Preference of adult chinchillas for low or high cages at different times of day.

During the light period, 13% of the chinchillas preferred the large cage, while during the dark period, 23% preferred the large cage. The majority of the chinchillas chose the small cages for resting. Even during their active periods, the small cages were chosen about three times more frequently than the large ones (*p* < 0.001). Preference for small cages peaked during the light phase (between 6:00 and 16:00), reaching nearly 90% (Figure 9). After the beginning of the dark period, there was a decline in preference for small cages, reaching approximately 70% around midnight. The opposite trend can be observed in the choice of the large cage (Figure 8).

### 3.2. Preference for Cages with Different Heights

The chinchillas were given a choice between a low cage (0.4 m high) and a high cage (1.0 m high), both with the same floor area.

#### 3.2.1. Floor Area: 0.25 m^2^

The chinchillas preferred the low cage 69% of the time, compared to 31% for the high cage, during the five-day monitoring period. There was no significant difference or tendency in preferences between days 1 and 5.

Adult chinchillas showed a clear preference for the low cage during the light phase (81%) compared to the dark phase (62%) (*p* < 0.001). The highest preference for the low cage was observed between 7:00 and 15:00 (about 80%), while this value decreased to 50% after midnight and then rose again (Figure 9).

#### 3.2.2. Floor Area: 0.5 m^2^

In the cage with a 0.25 m^2^ floor area, the chinchillas showed a preference for the low cage 70% of the time, compared to 30% for the high cage (*p* < 0.001). This preference remained consistent across different days, with 66–73% of the chinchillas preferring the low cage and 27–34% preferring the high cage.

**Figure 10:**
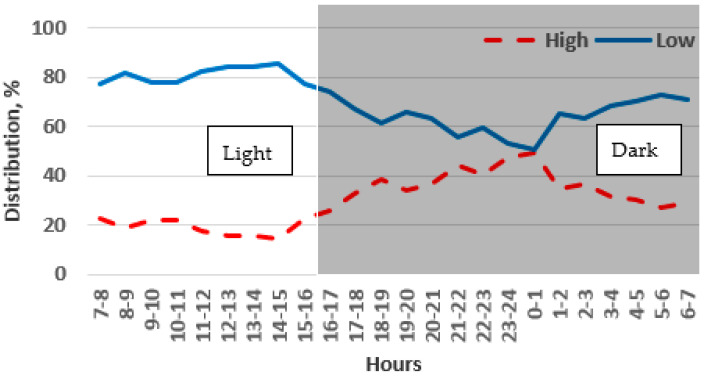
Preference of adult chinchillas for low or high cages based on time of day.

The chinchillas showed a stronger preference for the low cage during the light phase (81%) than during the dark phase (64%). This indicates that they preferred the low cage, particularly for resting. The highest preference for the low cage (80–85%) was observed between 8:00 and 15:00, with a decrease to 50% around midnight. Afterward, the preference for the low cage gradually increased again (Figure 10).

### 3.3. Cages with Different Floor Area and Height

After examining how changes in floor area and height independently affected chinchilla preferences, we then investigated how preferences changed when both floor area and height were increased simultaneously.

It was clear that the chinchillas preferred staying in the small-low (traditional) cages over the large-high ones. They chose the small-low cages 76% of the time, compared to 24% for the large-high cages (*p* < 0.001). No significant differences were found in preferences across observation days.

**Figure 11:**
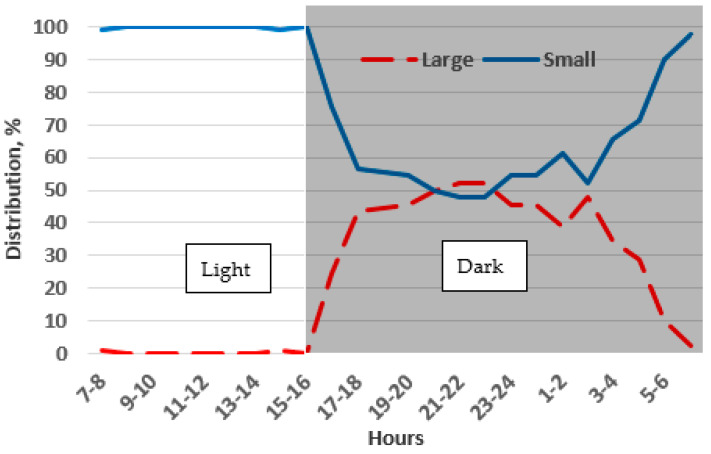
Preference of adult chinchillas between small-low (Small) and large-high cages (Large) based on time of day.

During the light phase of the day, the chinchillas spent 1% of their time in the large-high cages and 99% in the small-low cages, indicating a preference for the small-low cages while resting. However, during the dark phase, they spent 39% of their time in the large-high cages and 61% in the small-low cages, showing that they still favored the small-low cages, albeit less frequently, even when active. The preference for the small-low cages was nearly 100% during the light phase, but during the dark phase their preference between the small-low and large-high cages was sometimes nearly equal (50%) (Figure 11).

## 4. Discussion

This study is one of the few attempts to investigate cage size preferences in chinchillas. There are no previous results available for direct comparison. However, research conducted on other nocturnal prey species, particularly rabbits, provides useful insights. Like chinchillas, rabbits are prey animals that exhibit similar behavioral patterns, such as nocturnal activity and a tendency to seek shelter during the day. In the wild, rabbits live in colonies, signaling danger to each other and retreating to underground burrows to avoid predators, and they sleep there during the day [14,15,16,17]. On farms, adult rabbits are typically housed individually, while younger rabbits are housed in smaller or larger groups [18,19].

In our study, we found that the chinchillas showed a preference for smaller cages over larger ones, whether it was in terms of floor area or height. The chinchillas were observed 2.1 times more often in the smaller cages. This preference was even more evident in taller cages, where the chinchillas chose the lower cages 4.3 times more frequently. Throughout the day, the preference for smaller cages remained consistent. The chinchillas stayed in the smaller cages 2.7 times more frequently during the resting (light) period and 2.1 times more frequently during the active (dark) period. In the taller cages, the difference was even greater, with the chinchillas staying 6.7 times more frequently in the lower cages during the resting period and 6.1 times more frequently during the active period. These findings clearly demonstrate that the chinchillas, regardless of the time of day, prefer smaller cages over larger ones.

In studies involving rabbits, similar patterns have been noticed, although the variations are not as pronounced. For instance, Mikó et al. [20] discovered that, initially, rabbits spent more time in smaller cages, but this preference decreased over time. During the light period, rabbits showed a stronger inclination toward smaller cages, whereas, during the dark period, the difference in preference between smaller and larger cages was less notable. In another study, it was also noted that young rabbits favored smaller cages after weaning, and rabbit density in different-sized cages equalized with age [21].

In studies involving laboratory rodents such as rats and mice, there have been varying results regarding cage size preferences. While larger cages have been recommended [22], some studies have shown that smaller cages can reduce stress indicators in rats. For example, Sharp et al. [23] found that rats housed in smaller cages exhibited lower heart rates, blood pressure, and nocturnal activity, suggesting that they were less stressed than rats in larger cages. Similarly, in another study it was found that increasing cage size led to more anxiety-like behaviors in subordinate rats, indicating that a larger floor area does not always equate to better welfare [24]. Bailoo et al. [25] reported that cage size had little impact on welfare measures in mice, as indicated by unchanged glucocorticoid metabolite concentrations, while other researchers noted that smaller cages did affect some aspects of mouse behavior, such as play and posture [26].

The findings indicate that larger cages may not always lead to clear benefits. Our study showed that increasing the floor area did not result in a higher preference for it. Surprisingly, the chinchillas consistently preferred the smaller cages.

When it comes to cage height, the chinchillas preferred lower cages. The chinchillas choose lower cages 2.2 to 2.3 times more often than higher cages. This preference was more pronounced during the resting (light) period when the chinchillas selected lower cages 3.5 to 4.3 times more frequently. In the dark (active) period, the preference for lower cages was still evident, though less pronounced (1.6 to 1.8 times more frequent). These results align with studies on rabbits, where growing and adult rabbits showed a preference for lower or more enclosed spaces, likely because such environments offer a greater sense of security. It was found that rabbits chose the lowest cages more frequently and, when given the option, they often sought refuge under elevated platforms, which provided a sense of safety [27]. Other researchers also observed that rabbits preferred to stay in areas with lower tops, likely because these areas mimicked natural burrows [28].

In the present study, it was found that when both the floor area and the height of the chinchilla cages were changed, the chinchillas strongly preferred smaller and lower cages over larger and higher ones. During their resting period, very few chinchillas chose the larger cages, with only 1% of their time spent there. While, during the active period, the chinchillas showed a more balanced preference but still favored smaller cages 62% of the time. These results suggest that increasing both the floor area and height of cages does not provide any advantage for chinchilla housing.

The findings of rabbit studies suggest that cage size has little impact on reproductive performance or behavior. A Dutch research group compared standard cages to larger, double-sized cages and found no significant differences in production performances [29]. French researchers also found no behavioral or reproductive differences between rabbits housed in different-sized cages [30]. Although Bignon et al. [31] noted that female rabbits in larger cages were more active, this increased activity did not translate into significant reproductive benefits. Prola et al. [32] observed higher stress levels in rabbits housed in smaller cages, as indicated by increased corticosterone levels, suggesting that cage size can impact stress. However, in a review article, authors argued that instead of increasing cage size, environmental enrichments, such as elevated platforms, may provide greater benefits for animal welfare by allowing for more mobility and creating a sense of security [33].

Chinchillas, much like rabbits, are prey animals that feel safer in smaller, enclosed spaces. In the wild, chinchillas live in rocky crevices or burrows to avoid predators, and chinchillas on farms also exhibit similar behavior, preferring smaller cages that likely provide a greater sense of security [10]. This behavior suggests that larger cages may not be necessary or beneficial for chinchillas in captivity. As Baumans [34] pointed out, structuring the cage environment may be more important than increasing the floor area. Future research should focus on environmental enrichments to improve the welfare of chinchillas, rather than just increasing the cage size.

## 5. Conclusions

The study findings suggest that adult chinchillas show a preference for smaller and shorter cages when the small and large cages are not environment enrichment barren, especially during the rest period of the day, even when the floor area, height, or both are increased. It is recommended that future research focuses on exploring environmental enrichments to enhance the welfare of captive chinchillas, rather than just increasing cage dimensions.

## Data Availability

The original contributions presented in this study are included in the article.

## References

[1] Spotorno A.E., Zuleta C.A., Valladares J.P., Deane A.L., Jimenez J.E. (2004). *Chinchilla laniger* in Mammalian species. ASM.

[2] Valladares P., Zuleta C., Spotorno A. (2014). *Chinchilla lanigera* (Molina 1782) and *C. chinchilla* (Lichtenstein 1830): Review of their distribution and new findings. Anim. Biodivers. Conserv..

[3] Roach N., Kennerley R. (2016). Chinchilla lanigera (errata version published in 2017). The IUCN Red List of Threatened Species.

[4] Bidlingmaier T.C. (1937). Notes on the genus Chinchilla. J. Mammal..

[5] Jiménez J.E. (1996). The extirpation and current status of wild chinchillas *Chinchilla lanigera* and *C. brevicaudata*. Biol. Conserv..

[6] Parker W.D. (1975). Modern Chinchilla Farming.

[7] Heffer R.S., Heffer H.E. (1991). Behavioral hearing range of the chinchilla. Hear. Res..

[8] Shimoyama M., Smith J., De Pons J., Tutaj M., Khampang P., Hong W., Erbe C., Ehrlich G., Bakaletz L., Kerschner J. (2016). The chinchilla research resource database: Resource for an otolaryngology disease model. Database.

[9] Dzierżanowska-Góryń D., Kaleta T., Kowalczyk M. (2005). The behaviour and an activity of chinchilla (*Chinchilla lanigera*) kept under laboratory conditions. XIIth International Congress in Animal Hygiene (ISAH).

[10] Łapiński S., Niedbała P., Markowska K., Rutkowska A., Lis M.W. (2023). The effects of age, size, and cage complexity on the behaviour of farmed female chinchillas (*Chinchilla lanigera*). Sci. Rep..

[11] CoE Standing Committee of the European Convention for the Protection of Animals Kept for Farming Purposes (T-AP) (1999). Recommendation Concerning Fur Animals, Adopted by the Standing Committee.

[12] Łapiński S., Lis M.W., Wójcik A., Migdał Ł., Guja I. (2014). Analysis of factors increasing the probability of fur chewing in chinchilla (*Chinchilla lanigera*) raised under farm conditions. Ann. Anim. Sci..

[13] Łapiński S., Orel J., Niedbała P., Kucharska W., Jakubowska M., Lisowska-Lis A., Tombarkiewicz B., Lis M.W. (2020). Infrared Thermography as an Indicator of Heat Loss in Fur-Chewing Chinchillas (*Chinchilla lanigera*). J. Appl. Anim. Welf. Sci..

[14] Kolb H.H. (1986). Circadian activity in the wild Rabbit (*Oryctolagus cuniculus*). Mammal Rev..

[15] Cowan D.P. (1987). Group living in the European rabbit (*Oryctolagus cuniculus*): Mutual benefit or resource localization?. J. Anim. Ecol..

[16] Myers K., Parer I., Richardson B.J., Walton D.W., Richardson B.J. (1989). 45. Leporidae. Fauna of Australia—Volume 1b Mammalia.

[17] Palomares F. (2003). Warren building by European rabbits (*Oryctolagus cuniculus*) in relation to cover availability in a sandy area. J. Zool. Lond..

[18] Szendrő Z.S., Dalle Zotte A. (2011). Effect of housing conditions on production and behaviour of growing meat rabbits: A review. Livest. Sci..

[19] Szendrő Z.S., Trocino A., Hoy S.T., Xiccato G., Villagrá A., Maertens L. (2019). A review of recent research outcomes on the housing of farmed domestic rabbits: Reproducing does. World Rabbit Sci..

[20] Mikó A., Szendrő Z.S., Matics Z.S., Radnai I., Odermatt M., Nagy I., Gerencsér Z.S. (2012). Location preference of rabbit does between common sized and double sized cages. Acta Argic. Slov..

[21] Matics Z.S., Szendrő Z.S., Bessei W., Radnai I., Biró-Németh E., Orova Z., Gyovai M. The free choice of rabbits among identically and differently sized cages. Proceedings of the 8th World Rabbit Congress.

[22] National Research Council (2011). Guide for the Care and Use of Laboratory Animals.

[23] Sharp J., Azar T., Lawson D. (2003). Does cage size affect heart rate and blood pressure of male rats at rest or after procedures that induce stress-like responses?. Contemp. Top..

[24] Barker T.H., George R.P., Howarth G.S., Whittaker A.L. (2017). Assessment of housing density, space allocation and social hierarchy of laboratory rats on behavioural measures of welfare. PLoS ONE.

[25] Bailoo J.D., Murphy E., Varholick J.A., Novak J., Palme R., Würbel H. (2018). Evaluation of the effects of space allowance on measures of animal welfare in laboratory mice. Sci. Rep..

[26] Gaskill B.N., Pritchett-Corning K.R. (2015). The effect of cage surface on behavior and reproduction in Crl:CD1(Icr) and C57BL/6NCrl laboratory mice. PLoS ONE.

[27] Princz Z., Radnai I., Biróné Németh E., Matics Z.S., Gerencsér Z.S., Nagy I., Szendrő Z.S. (2008). Effect of cage height on the welfare of growing rabbits. Appl. Anim. Behav. Sci..

[28] Szendrő Z.S., Matics Z.S., Odermatt M., Gerencsér Z.S., Nagy I., Szendrő K., Dalle Zotte A. (2011). Use of different areas of pen by growing rabbits depending on the elevated platforms’ floor-type. Animal.

[29] Rommers J.M., Meijerhof R. (1998). La dimensions de la cage influence-t-elle la productivitè et la bien-èntre des lapins. Cuniculture.

[30] Mirabito L., Galliot P., Souchet C., Dumont F., Thomeret F. Logement collectif des lapines reproductrices: Conséquences zootechniques. Proceedings of the 11èmes Journées de la Recherche Cunicole.

[31] Bignon L., Bouchier M., Coutelet G., Galliot P., Souchet C., Fortun-Lamothe L. Individual housing of young does in different sized cages: Impact on welfare, economic costs and productive data. Proceedings of the 10th World Rabbit Congress.

[32] Prola L., Cornale P., Renna M., Macchi E., Perona G., Mimosi A. (2013). Effect of breed, cage type, and reproductive phase on fecal corticosterone levels in doe rabbits. J. Appl. Anim. Welf. Sci..

[33] Szendrő Z.S., McNitt I.J., Matics Z.S., Mikó A., Gerencsér Z.S. (2016). Alternative and enriched housing systems for breeding does: A review. World Rabbit Sci..

[34] Baumans V. (2005). Science-based assessment of animal welfare: Laboratory animals. Rev. Sci. Tech. OIE.

